# ﻿Four new species of *Trichoderma* (Hypocreaceae, Hypocreales) discovered in the staple food bamboo of pandas

**DOI:** 10.3897/mycokeys.124.163233

**Published:** 2025-11-03

**Authors:** Feihu Wang, Xiulan Xu, Feng Liu, Shasha Xiang, Xinyue Li, Yinggao Liu, Chunlin Yang

**Affiliations:** 1 College of Forestry, Sichuan Agricultural University, Chengdu, Sichuan, 611130, China Sichuan Agricultural University Chengdu China; 2 Forest Ecology and Conservation in the Upper Reaches of the Yangtze River Key Laboratory of Sichuan Province, Chengdu 611130, Sichuan Province, China Forest Ecology and Conservation in the Upper Reaches of the Yangtze River Key Laboratory of Sichuan Province Chengdu China; 3 Sichuan Mt. Emei Forest Ecosystem National Observation and Research Station, Chengdu 611130, Sichuan Province, China Sichuan Mt. Emei Forest Ecosystem National Observation and Research Station Chengdu China; 4 Forestry Research Institute, Chengdu Academy of Agricultural and Forestry Sciences, Chengdu 611130, Sichuan Province, China Forestry Research Institute, Chengdu Academy of Agricultural and Forestry Sciences Chengdu China

**Keywords:** Morphology, multi-gene, pandas, phylogenetic analyses, staple food bamboo, taxonomy, *
Trichoderma
*

## Abstract

*Trichoderma* fungi are significant saprophytic resources in nature, with only a minority of species documented as pathogenic fungi. Due to their widespread distribution, this genus of fungi has attracted considerable attention in recent years. During an investigation of fungal resources within the staple food bamboo species for giant pandas in China conducted from 2023 to 2024, a high diversity of *Trichoderma* species was observed. In this study, eight collected specimens were compared morphologically with known species, and DNA sequence analysis was performed using a multi-gene (ITS, *tef*1-α, and *rpb*2) dataset to establish phylogenetic relationships, ultimately leading to the identification of four *Trichoderma* species. The research uncovered four novel *Trichoderma* species: *Trichoderma bashania*, *T. fargesia*, *T. mianyangensis* and *T. yaanensis*. Phylogenetic analysis revealed that each of these new species forms a distinct lineage, with *Trichoderma bashania*, *T. fargesia*, *T. mianyangensis* and *T. yaanensis* all belonging to the Koningii section. All these newly identified species were isolated from the litter of the staple food bamboo species for giant pandas. This study provides morphological descriptions and illustrations of these four new species, along with DNA phylogenetic relationships based on the analysis of the multi-gene dataset. The findings indicate that *Trichoderma* fungi are widely present in the ecosystem of the staple food bamboo species for giant pandas and warrant close attention.

## ﻿Introduction

The giant panda (*Ailuropoda melanoleuca*), an endemic and rare species in China, is hailed as a “living fossil” and “China’s national treasure”. It also serves as the ambassador for the World-Wide Fund for Nature (WWF) and is a flagship species for global biodiversity conservation, as well as one of the most beloved wild animals worldwide (Ran et al. 2009). Although giant pandas are classified as carnivores and still retain the digestive system of carnivores, their diet is extremely narrow, with bamboo constituting the vast majority of their food intake. As a highly specialized species in terms of diet, bamboo resources account for over 99% of their food resources (Zhang et al. 2014). Based on fossil records, giant pandas have been exclusively bamboo-eaters for at least 2 million years. Scientists have never ceased their research into how giant pandas have adapted to a bamboo diet (Hansen et al. 2010; Senshu et al. 2014; He et al. 2020). However, not all bamboo species in nature can serve as food for giant pandas, and this singular food source makes giant pandas particularly vulnerable. Therefore, the study of the staple bamboo species for giant pandas is warranted (Tian et al. 2019). Currently, research on the staple bamboo species for giant pandas is limited to nutritional component analysis, while studies on microorganisms within the ecosystem of staple bamboo species remain largely unexplored (Helander et al. 2013; Jin et al. 2020). Research indicates that most plants are surrounded by a stable and complex microbial community, and changes in these microorganisms may trigger alterations in plants’ growth, development, health status, stress resistance, and other aspects. (Shang et al. 2024; Yang et al. 2024). Consequently, studying the fungi associated with the staple bamboo species for giant pandas is of great importance.

The genus *Trichoderma* (Hypocreaceae, Hypocreales, Sordariomycetes) was originally established by Persson in 1794. *Trichoderma* species are saprophytic fungi, commonly found in soil, wood, and litter (Hasan et al. 2012; Abo-Elyousr et al. 2014; Poveda et al. 2019; Zhao et al. 2023). Additionally, some *Trichoderma* species are significant pathogenic fungi, serving as key pathogens causing green mold disease and leading to the decay of a large number of cultivated mushrooms (Savoie and Mata 2003; Komon-Zelazowska et al. 2007; Tarafder et al. 2024). Finally, an increasing body of research indicates that *Trichoderma* species also function as important plant endophytes, playing crucial roles in the growth and development of plants (Lorito et al. 2010; Harman et al. 2021). Consequently, due to their economic and ecological significance, *Trichoderma* has evolved into a genus with a rich diversity of species. Currently, more than 500 species have been reported and recognized (http://www.indexfungorum.org). Meanwhile, species within this genus have been widely utilized, exerting a profound impact on humanity. They are efficient producers of bio-enzymes, important sources of biocontrol agents against plant pathogens, and key participants in environmental remediation, thus possessing extremely high economic value (Tripathi et al. 2013; Abdel-Mageed et al. 2022; Zhang et al. 2023).

Initially, the identification of *Trichoderma* relied solely on the different colors of ascospores in its sexual stage for differentiation (Rifai 1969). During its sexual stage, *Trichoderma* can produce ascospores of two distinct colors: hyaline (transparent) and green. Early research was based on these two-color variations for classification purposes (Bissett 1991). Chaverri and Samuels (2004) took the lead in conducting a comprehensive study on *Trichoderma* species with green ascospores. Although this provided early insights into the study of *Trichoderma*, the accuracy of identification was far from sufficient. Subsequently, Jaklitsch and Voglmayr (2015a) proposed an integrated classification system primarily based on molecular phylogenetic analysis rather than ascospore color, dividing the genus into six subclades: the Ceratocladosporium subclade, the Viride subclade, the Harzianum subclade, the Helicum subclade, the Asperellum subclade, and the Strictipile subclade. Currently, the identification of *Trichoderma* is gradually becoming standardized. The widespread use of DNA barcoding, such as the *rpb*2 and *tef*1-α coding genes, for identification purposes has enhanced the accuracy and standardization of the process (Cai and Druzhinina 2021; Zheng et al. 2021).

Species of the genus *Trichoderma* are extensively distributed across most regions worldwide, with their survival range spanning high-latitude areas from the far north to the far south. They demonstrate remarkable ecological adaptability, with their presence traced in ecosystems ranging from humid tropical and subtropical rainforests to arid deserts, temperate grasslands, and even northern boreal ecosystems (Cai and Druzhinina 2021). The rich biodiversity exhibited by this group, along with the continuous discovery and exploration of novel resources within it, has successfully garnered the attention of numerous researchers globally. Similarly, China has also shown a high level of interest in this group. However, in previous studies, most species were isolated from soil, and the majority of research was reported based on cultured strains (Zhang et al. 2022; Zhao et al. 2021, 2025). In this study, three sexual morph specimens and one asexual morph specimen were collected from four staple bamboo species for giant pandas, namely *Bashania faberi*, *Fargesia qinlingensis*, *Phyllostachys sulphurea*, and *Bambusa emeiensis*. The phylogeny of the four species were reconstructed using the gene sequences of ITS, *tef*1-α, and *rpb*2. The results met the criteria for identifying new species of *Trichoderma* (Cai and Druzhinina 2021).

## ﻿Materials and methods

### ﻿Specimen collection and isolation

This study involved collecting litter samples from the staple bamboo species for giant pandas in China (Sichuan Province and Shaanxi Province) between 2023 and 2024. The samples were collected using plastic sealing bags and transported back to the laboratory for subsequent experiments. The samples were examined and sectioned using a Leica stereo microscope (EZ 4). Single spore isolation was performed following the method described by Chomnunti et al. (2014). Hand-sectioning techniques were employed to prepare slide specimens of the fruiting bodies. Morphological structures were photographed using an Olympus BX53 microscope. All measurements of structural components were conducted using Tarosoft Image Framework software (version IFW 0.97). The type specimens have been deposited at the Herbarium of Sichuan Agricultural University (SICAU) in Wenjiang, Sichuan, China. All live cultures are preserved at the Strain Collection of Sichuan Agricultural University (SICAUCC). Based on the method provided by Jayasiri et al. (2015) for obtaining facial login numbers (http://www.facesoffungi.org/), all strains in this study have been registered in the Fungi Index (2025, http://www.indexfungorum.org/).

### ﻿DNA extraction, PCR amplification and nucleotide sequencing

Using a sterile stainless-steel spoon, fungal hyphae were scraped from Potato Dextrose Agar (PDA) and placed into a centrifuge tube containing steel beads. The hyphae were then thoroughly ground using a freeze-grinding instrument. The ground fungal hyphae were subjected to total genomic DNA extraction using a novel rapid extraction kit for plant genomic DNA (Aidilai Biotechnology Company, China). Three genetic regions were amplified, namely the Internal Transcribed Spacer (ITS) rRNA, Translation Elongation Factor 1-alpha (*tef*1-α), and RNA polymerase II subunit (*rpb*2). The amplified genetic markers and their corresponding primers are listed in (Table 1). Polymerase Chain Reaction (PCR) was performed in a 25 µL reaction mixture containing 12.5 µL of MasterMix (Beijing, China), 9.5 µL of deionized water 1 µL of DNA template, and 1 µL of each forward and reverse primer. The amplification conditions are shown in (Table 2). The PCR products were sequenced by Qingke Biotechnology Co., Ltd. in China. The newly generated sequences have been deposited in GenBank (Table 3).

**Table 1.** Sequences of primers used in this study.

Gene markers	Primers	Sequences of Primers 5'-3'	References
ITS	ITS5	GGAAGTAAAAGTCGTAACAAGG	White et al. 1990
	ITS4	TCCTCCGCTTATTGATATGC	
*te*f1-α	728F	CATCGAGAAGTTCGAGAAGG	Carbone and Kohn 1999
	EF2	GGARGTACCAGTSATCATG	O’Donnell et al. 1998
*rpb*2	5F	GAYGAYMGWGATCAYTTYGG	Liu et al. 1999
	7CR	CCCATRGCTTGYTTRCCCAT	

**Table 2.** Primers and PCR protocols used in this study.

Gene markers	Primers	Optimized PCR Protocols	References
ITS	ITS5	94 °C 3 min; 35 cycles of 94 °C 30 s, 55 °C 50 s, 72 °C 1 min; 72 °C 10 min; 4 °C on hold	White et al. 1990
	ITS4		
*tef*1	728F	94 °C 3 min; 35 cycles of 94 °C 30 s, 55 °C 50 s, 72 °C 1 min; 72 °C 10 min; 4 °C on hold	Carbone and Kohn 1999
	EF2		
*rpb*2	5F	95 °C 5 min; 35 cycles of 95 °C 1 min, 52 °C 2 min, 72 °C 90 s; 72 °C 10 min; 4 °C on hold	Liu et al. 1999
	7CR		

**Table 3.** Specimen information and GenBank accession numbers of the sequences used in this study.

Species	Strain	GenBank Accession Numbers		
		ITS	*rpb2*	*tef1α*
*Trichoderma albofulvopsis* W.T. Qin & W.Y. Zhuang	9930T	/	KU529138	KU529127
*Trichoderma amoenum* Z.F. Yu & Y.F. Lv	YMF 1.06209T	/	MT052192	MT070146
*Trichoderma arenarium* F. Cai, M.Y. Ding & Druzhin	TUCIM 10301T	/	MT242310	MT242303
*Trichoderma asperellum* Samuels, Lieckf. & Nirenberg	GJS 04-217	DQ381957	DQ333564	DQ381958
*Trichoderma atroviride* P. Karst	GJS 02-134	DQ315466	/	DQ307547
*Trichoderma austrokoningii* Samuels & Druzhin	GJS 99-146T	DQ323423	DQ367716	DQ307561
***Trichoderma bashania* Feihu Wang & C.L. Yang**	**SICAU 25-0179**	**PV789471**	**PV828316**	**PV828324**
***Trichoderma bashania* Feihu Wang & C.L. Yang**	**SICAU 25-0180T**	**PV789472**	**PV828317**	**PV828325**
*Trichoderma caribbaeum* Samuels & Schroers	GJS 97-3T	DQ313131	DQ328607	DQ284977
*Trichoderma changiae* Y.H. Wei & S.S. Tzean	BCRC 24F0002T	PP265989	PP273695	PP273697
*Trichoderma dingleyae* Samuels & Dodd	GJS 02-50T	DQ333548	DQ367718	DQ284978
*Trichoderma dorothopsis* A.A. Tomah & J.Z. Zhang	HZA5ET	/	MH647795	MK850827
*Trichoderma erinaceum* Bissett, C.P. Kubicek & Szakács	DIS 8	DQ313147	DQ323450	DQ284970
***Trichoderma fargesia* Feihu Wang & C.L. Yang**	**SICAU 25-0185T**	**PV789477**	**PV828320**	**PV828328**
***Trichoderma fargesia* Feihu Wang & C.L. Yang**	**SICAU 25-0186**	**PV789478**	**PV828321**	**PV828329**
*Trichoderma hamatum* (Bonord.) Bainier	DAOM 167057T	Z48816	DQ111962	AF456911
*Trichoderma hongkuii* C.L. Zhang	GDMCC 3.1017T	/	OR779477	OR779504
*Trichoderma istrianum* Jaklitsch & Voglmayr	CBS 130539T	/	KJ665281	KJ665523
*Trichoderma intricatum* Samuels & Dodd	GJS 97-88T	AY380913	AY376684	AY376060
*Trichoderma koningii* Oudem	CBS 457.96T	Z79628	DQ341180	AF456909
*Trichoderma koningiopsis* Samuels, Carm. Suárez & H.C. Evans	GJS 93-20	/	DQ381954	DQ284966
*Trichoderma merleae*merleae Y.P. Tan, Minns, Valter, Marney & E. Lacey	MST FP3586T	PQ570877	PQ572711	PQ572712
***Trichoderma mianyangensis* Feihu Wang & C.L. Yang**	**SICAU 25-0183T**	**PV789475**	**PV828322**	**PV828330**
***Trichoderma mianyangensis* Feihu Wang & C.L. Yang**	**SICAU 25-0184**	**PV789476**	**PV828323**	**PV828331**
*Trichoderma neohongkuii C.L. Zhang*	GDMCC 3.1018T	/	OR779481	OR779508
*Trichoderma ovalisporum* Samuels & Schroers	DIS 70aT	/	AY376671	AY376037
*Trichoderma parahamatum* C.L. Zhang	GDMCC 3.1020T	/	OR779474	OR779501
*Trichoderma parahongkuii* C.L. Zhang	GDMCC 3.1019T	/	OR779476	OR779503
*Trichoderma petersenii* Samuels, Dodd & Schroers	GJS 04-355T	/	DQ333570	DQ284980
*Trichoderma rogersonii* Samuels	GJS 04-158T	/	DQ333567	DQ307563
*Trichoderma stilbohypoxyli* Samuels & Schroers	CBS 992.97T	/	DQ111967	DQ109546
*Trichoderma taiwanense* Samuels & M.L. Wu	GJS 95-93T	DQ313141	/	DQ284973
*Trichoderma viride* Pers	CBS 433.34	AF456922	/	AF456905
*Trichoderma gamsii* Samuels & Druzhin	GJS 04-09	DQ315459	/	DQ307541
*Trichoderma vinosum* Samuels	GJS 02-54	DQ315447	/	DQ307528
*Trichoderma viride* Pers	GJS 04-353	DQ323418	/	DQ307551
*Trichoderma texanum* Q.V. Montoya, L.A. Meirelles, P. Chaverri & A. Rodrigues	LESF551T	HQ608136	KT278920	KT278988
*Trichoderma tibetica* Z.F. Yu & X. Du	YMF 1.05583T	MK779177	MK779178	MK779179
***Trichoderma yaanensis* Feihu Wang & C.L. Yang**	**SICAU 25-0181T**	**PV789473**	**PV828318**	**PV828326**
***Trichoderma yaanensis* Feihu Wang & C.L. Yang**	**SICAU 25-0182**	**PV789474**	**PV828319**	**PV828327**

Notes: superscript T represents ex-type or ex-epitype isolates. “/” means that the sequence is missing or unavailable. The new sequence is displayed in bold red.

### ﻿Sequence alignment and phylogenetic analyses

A phylogenetic analysis of the genus *Trichoderma* was conducted based on ITS, *tef*1-α, and *rpb*2 sequence data. In this study, the strain *T. hamatum* (DAOM 167057) and *T. asperellum* (GJS 04-217) were selected as the outgroup for phylogenetic analysis. The sequences generated in this study were examined and assembled using BioEdit v.7.0.9 (Hall 1999). Sequence alignment was performed using MAFFT v.7 online software (https://mafft.cbrc.jp/alignment/server/) (Katoh and Standley 2013). Based on the combined dataset, phylogenetic analyses were conducted using Maximum Likelihood (ML) and Bayesian Inference (BI), were constructed as described in Zhao et al. (2025). The phylogenetic trees were viewed using FigTree v1.4.3 (Rambaut and Drummond 2016) and edited using Adobe Illustrator 2021 (version 2.6.0.44) and Adobe Photoshop CS6 software (Adobe Systems, USA).

### ﻿Genealogical concordance phylogenetic species recognition analysis

Genealogical Concordance Phylogenetic Species Recognition (GCPSR) is a pivotal and unique method in the field of biological research for model validation. It can efficiently and precisely detect significant recombination events within datasets (Taylor et al. 2000). In this study, we employed the powerful SplitsTree V4 software and conducted an in-depth analysis of the data using the pairwise homoplasy index (PHI) test. This test meticulously examines genetic similarities, enabling us to accurately determine the recombination levels among closely related species. When the pairwise homoplasy index falls below the threshold of 0.05 (Φw < 0.05), it clearly indicates the presence of significant recombination events in the dataset. To ensure the accuracy and comprehensiveness of the analysis results, the study selected a concatenated multi-gene dataset encompassing all closely related species. This comprehensive dataset provides a more reliable genetic foundation for the research. Based on this dataset, a split graph was constructed using the LogDet transformation and split decomposition options to visually present the complex genetic relationships.

## ﻿Results

### ﻿Phylogenetic analyses

**Figure 1.**
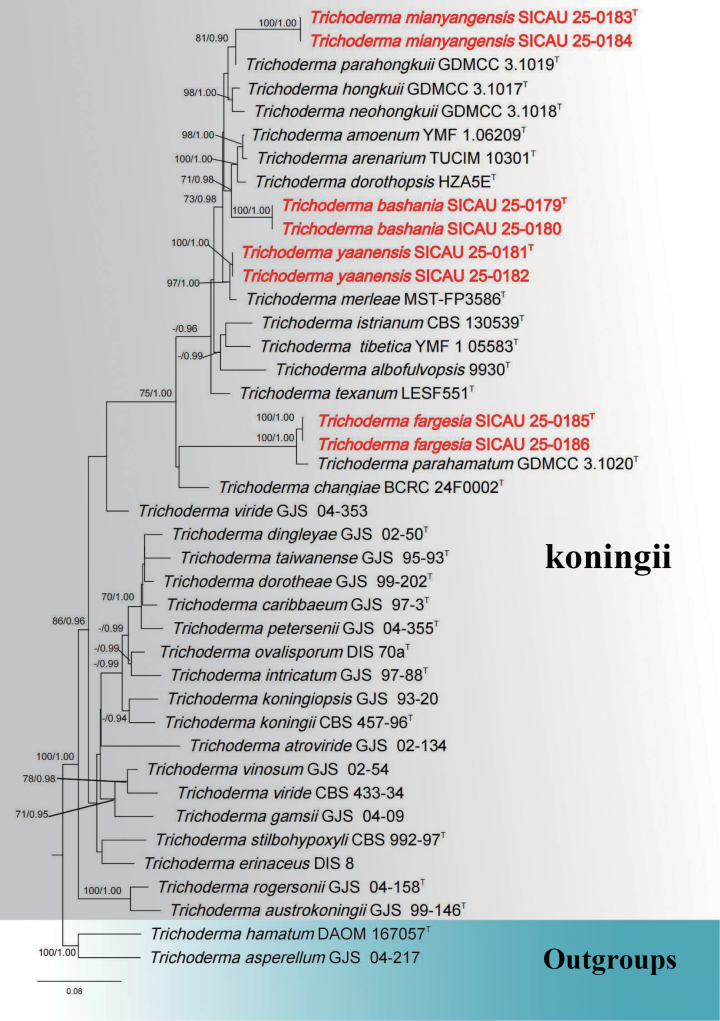
Phylogram generated from maximum likelihood analysis based on combined ITS, *tef*1-α and *rpb*2 sequence data of *Trichoderma* (koningii species complex) taxa. The species determined in this study are indicated in bold red. Bootstrap values (BS) from maximum likelihood (MLBS, left) higher than 70 BS and Bayesian posterior probabilities (BYPP, right) greater than 0.90 are given at the nodes. Hyphens (-) represent support values less than 70 MLBS/0.90 BYPP. The ex-type strains are in T.

This dataset comprises composite sequences formed by concatenating ITS, *tef*1-α and *rpb*2 gene sequences. These sequences are employed to elucidate and determine the phylogenetic position of the new species within the genus *Trichoderma*. The phylogenetic tree was performed for placement determination. 41 strains were included in the combined analyses, which comprised 3705 characters (1831 characters for *tef*1-α, 672 characters for ITS and 1202 characters for *rpb*2, including alignment gaps). The tree was rooted with *T. hamatum* DAOM 167057 and *T. asperellum* GJS 04-217. The best scoring RAxML tree had a fnal likelihood value of -16267.318033. The matrix had 1213 distinct alignment patterns, with 47.89% of undetermined characters and gaps. Estimated base frequencies were: A = 0.228333, C = 0.285248, G = 0.235065, T = 0.251354; substitution rates AC = 1.085759, AG = 2.939729, AT = 1.067672, CG = 0.871080, CT = 4.506612, GT = 1.000000; gamma distribution shape parameter α = 0.458789. The Bayesian inference analyses was implemented by MrBayes v.3.2.2 with the best-fit model (HKY+I+G for tef1-α; GTR+I+G for ITS and rpb2) of evolution estimated with MrModeltest 2.2. The maximum likelihood (ML) and Bayesian methods (BI) for phylogenetic analyses resulted in trees with similar topologies, and the result of ML analysis is shown in Fig. 1.

### ﻿Taxonomy

#### ﻿ Trichoderma bashania

Taxon classificationFungiHypocrealesHypocreaceae

Feihu Wang & C.L. Yang sp. nov.

C2CC0FD8-7E19-5EAF-8E27-3B0B8C35EDF4

Index Fungorum number: IF904040

Fig. 3

##### Etymology.

Named after the genus of the host plant from which the holotype was collected, *Bashania faberi*.

##### Holotype.

SICAU 25-0179.

##### Habitat.

On the culm of *Bashania faberi*.

##### Description.

***Sexual morph***: ***Stromata*** scattered, sparsely spreading, growing on the side adjacent to the ground surface, base narrow, pulvinate or discoidal. Color of stromata pale orange-yellow when fresh, brown when mature, with a diameter of 1–4 mm and a thickness of 1–2 mm. Immature, surface of stromata finely velvety, upon maturity, tuberculate with fine granules or slightly rugose. Outline circular, oblong or irregularly lobed. Surface smooth, tubercular or rugose, when young finely velvety. ***Ascomata*** 120–190 × 80–140 μm (x– = 145 × 115 μm, n = 30), numerous, with 10–30 ascomata present, and are sub-globose or pear-shaped in form. ***Ostioles*** flush with the surface, 18–30 μm wide at the apex, 26–38 μm high (n = 20). ***Peridium*** 13–24 μm (n = 60) thick at the base, composed of hyaline textura globosa. ***Asci*** 60–90 × 3.5–6 μm (x– = 85 × 4.5 μm, n = 30), short stipe, containing 13-ascospores, apex not thickened, hyaline, cylindrical. ***Ascospores*** 3–6 × 2.5–4.5 μm (x– = 4.5 × 3 μm, n = 50), hyaline, containing 1–2 oil droplets, single-celled, non-septate, and sub-globose. ***Asexual morph***: ***Conidiophores*** simple structure, with 1–3 solitary phialides borne at the tips of lateral branches. ***Phialides*** measure 4–18 × 1.5–3 μm (x– = 14 × 2.5 μm, n = 20), mostly lageniform, less commonly subfusiform, and typically do not thicken near the base. ***Conidia*** vary in size and shape, measuring 2.5–4.5 × 2–4 μm (x– = 4 × 3 μm, n = 50), oval, ellipsoid, and hyaline, smooth-surfaced.

##### Material examined.

China • Sichuan Province, Chengdu City, Dujiangyan, Primitive Forest of *Bashania faberi* (31°12'8.63"N, 103°42'49.96"E, Alt. 1212 m), 11 December 2023, Feihu Wang, WFH202312013, (SICAU 25-0179, holotype), ex-type culture SICAUCC 25-0151. *ibid*. WFH202312013B (SICAU 25-0180, paratype), living culture SICAUCC 25-0152.

##### GenBank accession numbers.

SICAUCC 25-0151 (ITS: PV789471; *tef*1-α: PV828324; *rpb*2: PV828316); SICAUCC 25-0152 (ITS: PV789472; *tef*1-α: PV828325; *rpb*2: PV828317).

##### Culture characters.

This fungal strain exhibits optimal growth on all tested media at 30 °C, whereas growth is restricted or completely inhibited at 35 °C. On PDA, at 30 °C, the colony slowly covers a 60-mm Petri dish within 10 days, displaying a grayish-white color with a radial growth pattern and fluffy texture. Conidiation initiates after 15 days, producing numerous white conidia that aggregate into irregularly margined patches. On SNA, growth is the slowest among the three media; at 30 °C, the colony reaches a radius of 50 mm after 10 days, remaining white with a radial pattern and sparse hyphae. Conidiation begins 15 days post-inoculation, forming white, irregularly margined conidial patches composed of aggregated conidia. No odor or pigment diffusion is detected. On MEA, at 30 °C, the colony rapidly covers the entire 60-mm dish within 7 days, featuring a well-defined border, radial growth, and creamy-white mycelium with dense, abundant aerial hyphae. No distinct odor or diffusing pigment is observed.

##### Notes.

**Figure 2.**
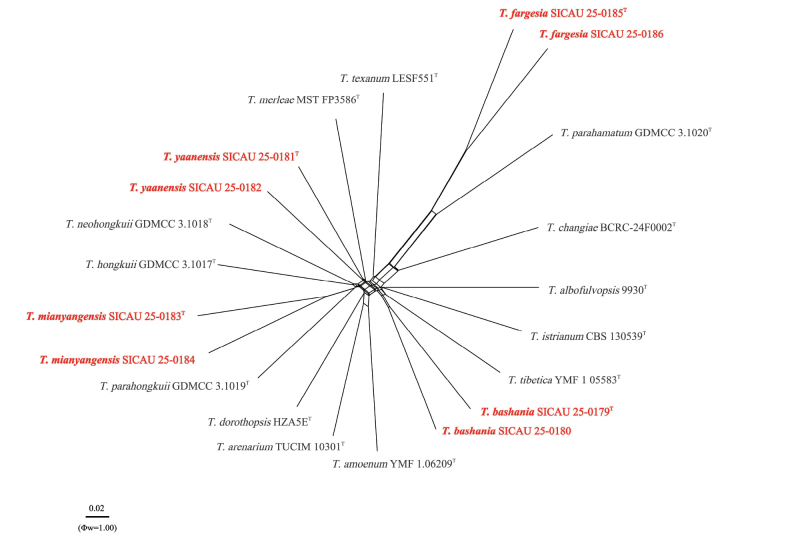
The PHI test results for *Trichoderma bashania*, *T. fargesia*, *T. mianyangensis*, *T. yaanensis*, and closely related species were obtained using both LogDet transformation and splits decomposition methods. The PHI test did not find any statistically significant recombination (Φw = 1) in the data set. The new strains are represented in red and bold. ^“T”^ represents the type strain.

Phylogenetically, *Trichoderma bashania* (SICAU 25-0179) and (SICAU 25-0179) formed a distinct clade and is related to *T. dorothopsis* (HZA5E) in the Koningii clade, but the similarities of *rpb*2 and *tef*1-α between these two species were only 95.3% and 98.3%, respectively. From a morphological perspective, only the asexual stage of *T. dorothopsis* has been described, and there are differences in conidia between these two *Trichoderma* species. The conidia of *T. bashania* are elliptical or ovoid, whereas those of *T. dorothopsis* are globose to subglobose (Tomah et al. 2020). Phylogenetic analysis indicates that the new taxon *T. bashania* (SICAU 25-0179) is closely related to *T. dorothopsis* (HZA5E) (Fig. 1). However, our strain exhibits nucleotide differences from *T. dorothopsis* in the *rpb*2 region amounting to 4.7% (39/822, 3 gaps), 1.7% (13/753, 0 gap) differences in *tef*1-α. The PHI test revealed no significant recombination event between our strain and the closely related taxa (Φw = 1.00) (Fig. 2). These differences also support the classification of *T. bashania* as a distinct species separate from *T. dorothopsis*.

**Figure 3.**
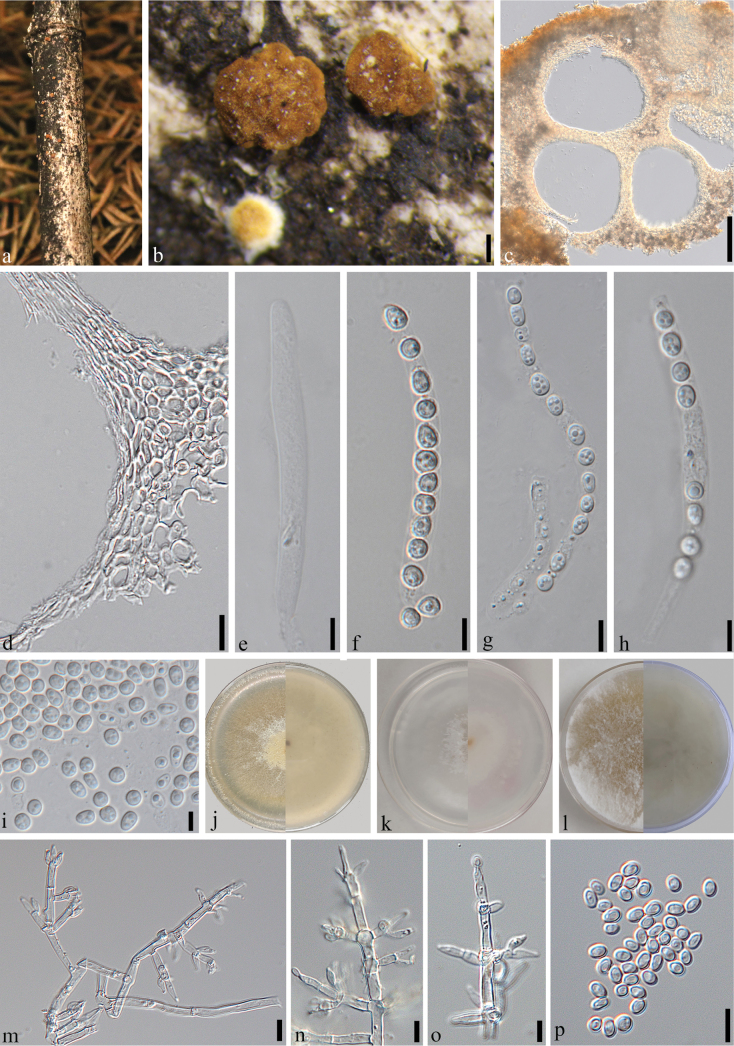
*Trichoderma bashania* (SICAU 25-0179, holotype). a. Appearance of ascomata on dead host; b. Stromata; c. Ascomatal tissue in section; d. Peridium; e–h. Asci; i. Ascospores; j. Culture on PDA (15 days at 30 °C); k. Cultures on SNA (15 days at 30 °C); l. Cultures on MEA (15 days at 30 °C); m–o. Conidiophores and phialides (PDA 30 days at 30 °C); p. Conidia (PDA 30 days at 30 °C). Scale bars: 500 µm (b); 200 µm (c); 10 µm (d–h, p); 5 µm (i, m–o).

#### ﻿ Trichoderma fargesia

Taxon classificationFungiHypocrealesHypocreaceae

Feihu Wang & C.L. Yang sp. nov.

8C7DF400-C9E5-573E-B50B-9B6CB6120630

Index Fungorum number: IF904041

Fig. 4

##### Etymology.

Named after the genus of the host plant from which the holotype was collected, *Fargesia qinlingensis*.

**Figure 4.**
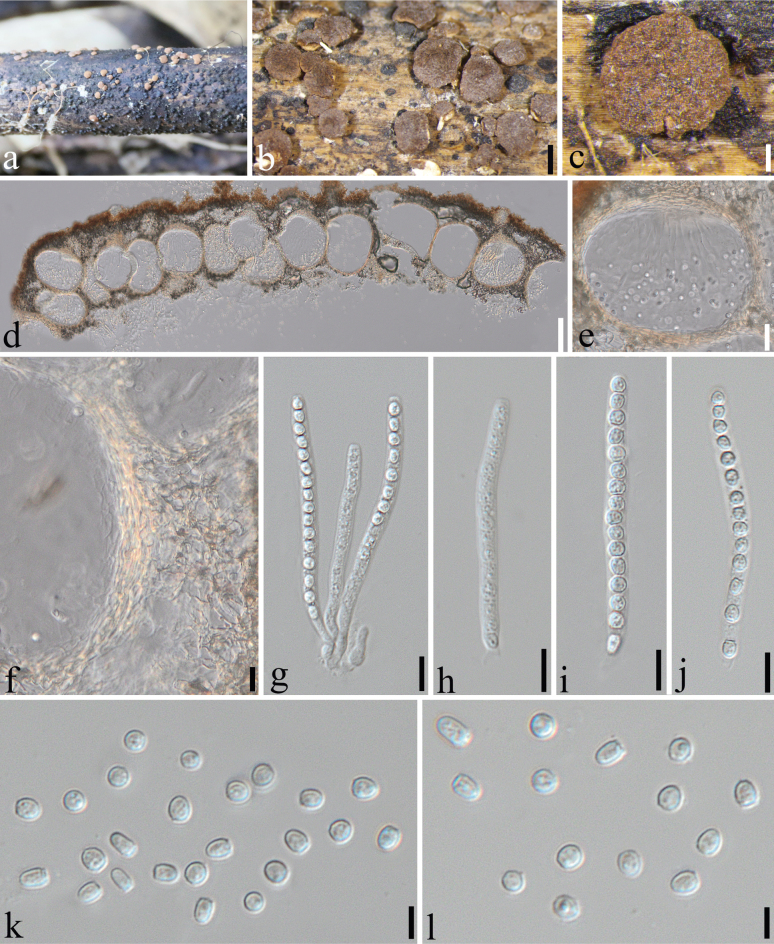
*Trichoderma fargesia* (SICAU 25-0185, holotype). a. Appearance of ascomata on dead host; b, c. Stromata; d, e. Ascomatal tissue in section; f. Peridium; g–j. Asci; k, l. Ascospores. Scale bars: 5 mm (b); 1 mm (c); 200 µm (d); 50 µm (e); 15 µm (f–j); 5 µm (k, l).

##### Holotype.

SICAU 25-0185.

##### Habitat.

On the culm of *Fargesia qinlingensis*.

##### Description.

***Sexual morph***: ***Stromata*** scattered or aggregated in small numbers, lenticular to pulvinate in shape. Centrally attached, free margins, rounded, angular, or irregular in outline. Color ranges from light reddish-brown to dark reddish-brown, with diameters of 2–8 mm and thicknesses of 1–3 mm (n = 20). Young stromata surface velvety, covered pale yellowish hairs. Later stages, surface finely granular from perithecial contours, appearing without any covering. Ostiolar dots on stromata inconspicuous. ***Ascomata*** nearly spherical and densely arranged, measuring 150–280 × 90–240 μm (x– = 220 × 175 μm, n = 20). ***Ostioles*** flush with the stroma surface, with apical widths of 34–50 μm and heights of 45–78 μm (n = 20). ***Peridium*** ranging from hyaline to brown, lateral thickness of 4–14 μm and a basal thickness of 8–18 μm (n = 20). ***Asci*** cylindrical, measuring 65–84 × 4–6 μm (x– = 75 × 5 μm, n = 30), inclusive of a stipe 7–12 μm long, containing 16-ascospores, apex not thickened, hyaline, cylindrical. ***Ascospores*** 3–5 × 2.5–4 μm (x– = 4.5 × 2.8 μm, n = 40) hyaline, nearly spherical to ellipsoidal. ***Asexual morph***: Not observed.

##### Material examined.

China • Shaanxi Province, Ankang City, Ningshan, Primitive Forest of *Fargesia qinlingensis* (33°35'45.67"N, 108°3'54.18"E, Alt. 2280 m), 15 October 2024, Feihu Wang, WFH202410014, (SICAU 25-0185, holotype). *ibid*. WFH202410014B (SICAU 25-0186, paratype).

##### GenBank accession numbers.

SICAU 25-0185 (ITS: PV789475; *tef*1-α: PV828328; *rpb*2: PV828320); SICAU 25-0186 (ITS: PV789476; *tef*1-α: PV828329; *rpb*2: PV828321).

##### Culture characters.

No germination was observed on potato dextrose agar (PDA), oatmeal agar (SNA), or malt extract agar (MEA).

##### Notes.

Phylogenetically, *Trichoderma fargesia* strain SICAU 25-0185 and SICAU 25-0186) formed a distinct clade and is related to *T. parahamatum* (GDMCC 3.1020) in the Koningii clade, but the similarities of *rpb*2 and *tef*1-α between these two species were only 86.2% and 90.5%, respectively. During the isolation and cultivation process, *T. fargesia* failed to germinate in sterile water and several culture media. *Trichoderma parahamatum* has only been described in its asexual stage, characterized by conidiophores that are coiled, undulate, or hamate, bearing phialides that are ampulliform to subglobose, short, and wide. Conidia are subglobose, ellipsoidal to breviter cylindracea, and green. Moreover, *T. parahamatum* is capable of germinating in several culture media. Phylogenetic analysis indicates that the new taxon *T. fargesia* (SICAU 25-0185) is closely related to *T. parahamatum* (GDMCC 3.1020) (Fig. 1). However, our strain exhibits nucleotide differences from *T. parahamatum* in the *rpb*2 region amounting to 13.8% (155/1116, 8 gaps), 9.5% (86/898, 6 gaps) differences in *tef*1-α. Pairwise nucleotide comparisons further support the distinction of *T. fargesia* from related taxa. The PHI test revealed no significant recombination event between our strain and the closely related taxa (Φw = 1.00) (Fig. 2). *Trichoderma fargesia* forms a distinct branch within the Koningii clade, leading to its identification as a new species.

#### ﻿ Trichoderma mianyangensis

Taxon classificationFungiHypocrealesHypocreaceae

Feihu Wang & C.L. Yang sp. nov.

7627F684-17F2-5586-8188-15C3A27B0E1F

Index Fungorum number: IF904042

Fig. 5

##### Etymology.

The specific epithet is derived from the collection locality of the type specimen, Mianyang City, Sichuan Province.

**Figure 5.**
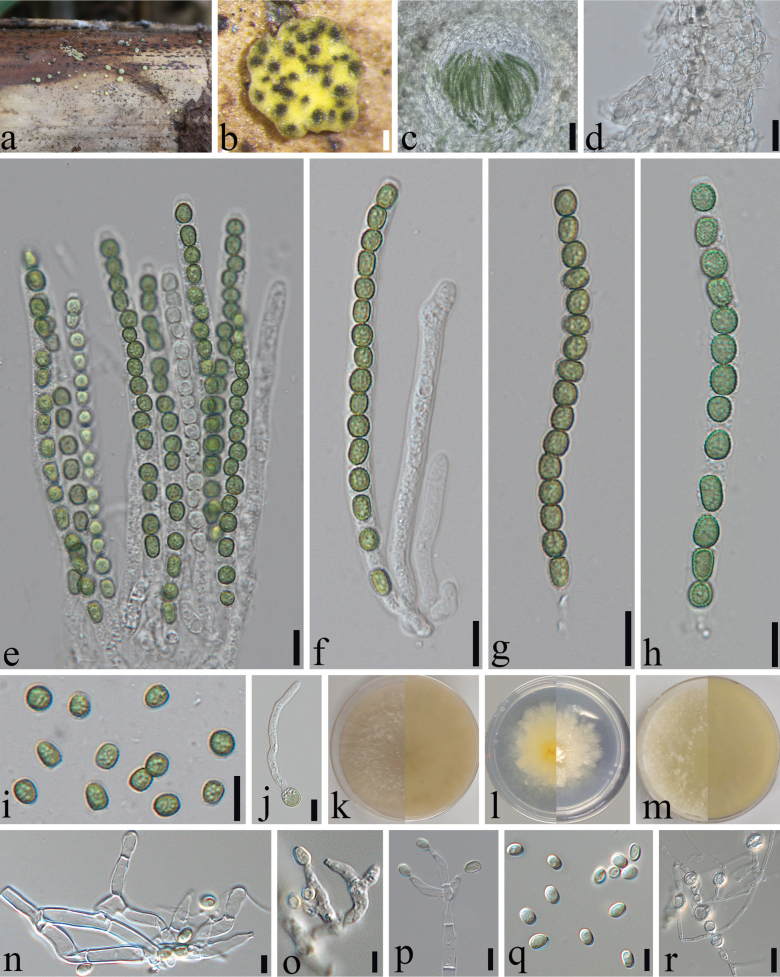
*Trichoderma mianyangensis* (SICAU 25-0183, holotype). a. Appearance of ascomata on dead host; b. Stromata; c. Ascomatal tissue in section; d. Peridium; e–h. Asci; i. Ascospores; j. Germinated ascospore; k. Culture on PDA (15 days at 25 °C); l. Cultures on SNA (15 days at 25 °C); m. Cultures on MEA (15 days at 25 °C); n–p. Conidiophores and phialides (PDA 30 days at 25 °C); q. Conidia (PDA 30 days at 25 °C); r. Chlamydospores (PDA 30 days at 25 °C). Scale bars: 500 µm (b); 50 µm (c); 10 µm (e–i, r); 5 µm (j, n–q).

##### Holotype.

SICAU 25-0183.

##### Habitat.

On the culm of *Phyllostachys sulphurea*.

##### Description.

***Sexual morph***: ***Stromata*** solitary, scattered, or aggregated in small numbers (2–3), presenting a pulvinate, discoid, lrregular in shape, broad-based in attachment. Ranging in color from light yellow to nearly citrine, with diameters of 3–10 mm and thicknesses of 1–3 mm (n = 30). Surface finely tuberculate or wrinkled. Margins rounded, angular, or undulate, typically free, sides slightly retracted inward. Ostiolar dots minute, distinct, surface rarely convex. ***Ascomata*** nearly spherical, densely arranged, and numerous, measuring 220–360 × 180–270 μm (x– = 290 × 170 μm, n = 20). ***Ostioles*** flush with the surface, with apical widths of 35–58 μm and heights of 40–78 μm (n = 20). ***Peridium*** hyaline to light yellow, with a lateral thickness of 8–12 μm and a basal thickness of 11–17 μm (n = 20). ***Asci*** 82–108 × 5–7 μm (x– = 100 × 6 μm, n = 20), inclusive of a stipe 9–20 μm long, cylindrical, containing 16-ascospores, apex slightly thickened, hyaline. ***Ascospores*** 3–6 × 3.5–4.5 μm (x– = 5 × 4 μm, n = 40), green, partially hyaline, finely spinulose, and ranging from nearly spherical to slightly ovoid in shape. ***Asexual morph***: ***Conidiophores*** consist of an erect stipe with a 1–3 celled branch at the apex. ***Phialide*** t lageniform, ampulliform, or subglobose, measuring 4–12 × 1.5–3 μm (x– = 9 × 2 μm, n = 30). ***Conidia*** hyaline, ellipsoidal to oblong, less commonly subglobose, smooth, and measure 2.5–4 × 1.5–3 μm (x– = 3 × 2 μm, n = 40).

##### Material examined.

China • Sichuan Province, Mianyang City, Pingwu, Primitive Forest of *Phyllostachys sulphurea* (32°37'13.81"N, 104°31'21.63"E, Alt. 1363 m), 18 October 2024, Feihu Wang, WFH20240092, (SICAU 25-0183, holotype), ex-type culture SICAUCC 25-0155. *ibid*. WFH20240092B (SICAU 25-0184, paratype), living culture SICAUCC 25-0156.

##### GenBank accession numbers.

SICAUCC 25-0155 (ITS: PV789477; *tef*1-α: PV828330; *rpb*2: PV828322); SICAUCC 25-0156 (ITS: PV789478; *tef*1-α: PV828331; *rpb*2: PV828323)

##### Culture characters.

Three media were tested for fungal growth: potato dextrose agar (PDA), oatmeal agar (SNA), and malt extract agar (MEA), all incubated at the optimal temperature of 25 °C. On PDA, the mycelium fully covered a 60-mm Petri dish within 8–10 days, forming a circular colony with a regular margin and velvety to floccose surface hyphae arranged radially. Abundant aerial hyphae extended toward the margin, becoming floccose post-conidiation. By day 12, conidiation initiated around the inoculation plug, forming small white clusters that expanded peripherally while remaining white. No diffusing pigment or distinct odor was detected. On SNA, growth was slower, requiring 20 days to fully colonize the dish (colony diameter: 20 mm after 12 days). The colony remained white and transparent, with sparse mycelium on the agar surface. Central mycelia became hollow, forming a loose, white film-like structure. No pigment or odor was observed, though the inoculation site gradually greened, conidia were absent. On MEA, rapid growth covered the dish within 7–9 days, yielding a circular, transparent colony with conspicuous variation in hyphal width (no zonation). Aerial hyphae were sparse initially but thickened at distal/lateral margins over time. By day 11, conidiation initiated on aerial hyphae near the proximal margin, forming fluffy tufts that compacted into 1–3 mm pustules with, pustules remained white for 5–8 days, then turned brown. No diffusing pigment or odor was detected.

##### Notes.

Phylogenetically, *Trichoderma mianyangensis* strain SICAU 25-0183 and SICAU 25-0184 formed a distinct clade and is related to *T. parahongkuii* (GDMCC 3.1019) in the Koningii clade, but the similarities of *rpb*2 and *tef*1-α between these two species were only 94.4% and 80.9%, respectively. From a morphological perspective, our newly described taxon, *Trichoderma mianyangensis*, shares some common characteristics with *T. parahongkuii* isolated from soil in a *Chimonanthus praecox* orchard. There are certain differences in cultural characteristics between *T. mianyangensis* and *T. parahongkuii*, differences in cultural characteristics exist between *T. mianyangensis* and *T. parahongkuii*. *Trichoderma mianyangensis* lacks any odor, while *T. parahongkuii* emits a noticeable fruity scent, and the conidia of *T. mianyangensis* are smaller (2.5–4 × 1.5–3 μm vs. 3.6–5.1 × 2.6–3.5 μm) (Zhao et al. 2025). Phylogenetic analysis reveals that the new taxon, *T. mianyangensis* (SICAU 25-0183), is closely related to *T. parahongkuii* (GDMCC 3.1019), with strong statistical support (Fig. 1). However, our strain differs from *T. parahongkuii* in the *rpb*2 region amounting to 5.6% (63/1130, 0 gap), 19.1% (178/930, 61 gaps) differences in *tef*1-α. Pairwise nucleotide comparisons further support the distinction of *T. mianyangensis*. The PHI test revealed no significant recombination event between our strain and the closely related taxa (Φw = 1.00) (Fig. 2). These differences also support the classification of *T. mianyangensis* as a distinct species.

#### ﻿ Trichoderma yaanensis

Taxon classificationFungiHypocrealesHypocreaceae

Feihu Wang & C.L. Yang sp. nov.

FF0D8F22-D29D-56C9-BC8E-24A0B6050999

Index Fungorum number: IF904044

Fig. 6

##### Etymology.

The specific epithet is derived from the collection locality of the type specimen, Ya’an City, Sichuan Province.

**Figure 6.**
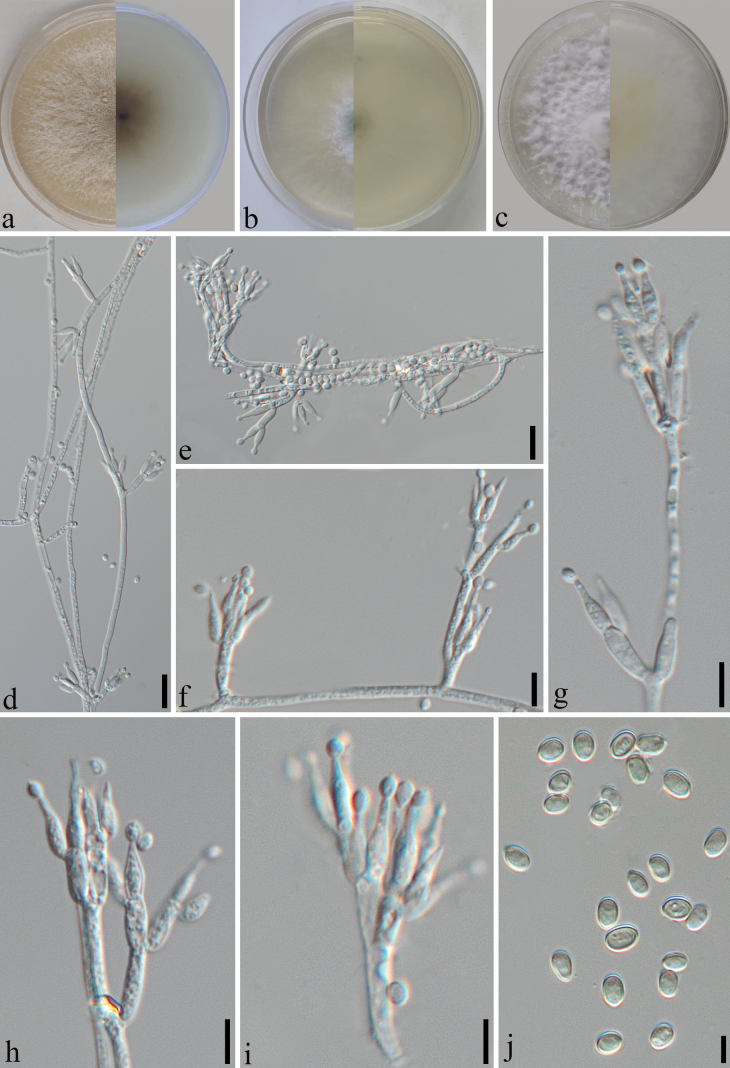
*Trichoderma yaanensis* (SICAU 25-0181, holotype). a. Culture on PDA (15 days); b. Cultures on SNA (15 days at 25 °C); c. Cultures on MEA (15 days at 25 °C); d–i. Conidiophores and phialides (PDA 30 days at 25 °C); j. Conidia (PDA 30 days at 25 °C). Scale bars: 20 µm (d, e); 15 µm (f); 10 µm (g–i); 5 µm (j).

##### Holotype.

SICAU 25-0181.

##### Habitat.

On the culm of *Bambusa emeiensis*.

##### Description.

***Sexual morph***: Not observed. ***Asexual morph***: ***Conidiophores*** are short and simple, either erect or arising obliquely from the surface hyphae. ***Phialides*** are solitary, whorls, and often borne on one-celled lateral branches, being conical or cylindrical in shape. ***Conidia*** 3.5–5.5 × 2–3.5 μm (x– = 4 × 2.6 μm, n = 40), pale green, smooth, ovoid or ellipsoidal.

##### Material examined.

China • Sichuan Province, Ya’an City, Yucheng, *Bambusa emeiensis* industrial base (29°52'44.35"N, 103°5'23.37"E, Alt. 871 m), 28 April 2024, Feihu Wang, WFH202404030, (SICAU 25-0181, holotype), ex-type culture SICAUCC 25-0153. *ibid*. WFH202404030B (SICAU 25-0182, paratype), living culture SICAUCC 25-0154.

##### GenBank accession numbers.

SICAUCC 25-0153 (ITS: PV789473; *tef*1-α: PV828326; *rpb*2: PV828318); SICAUCC 25-0154 (ITS: PV789474; *tef*1-α: PV828327; *rpb*2: PV828319).

##### Culture characters.

Three media were tested for fungal growth: potato dextrose agar (PDA), oatmeal agar (SNA), and malt extract agar (MEA), all incubated at the optimal temperature of 25 °C. On PDA, the mycelium fully covered a 60 mm Petri dish within 9–10 days, forming a circular, dense colony with a regular margin and sparse surface hyphae arranged radially. By day 15, small white velvety masses appeared, later browning and producing conidia; no odor or pigment was detected. On SNA, growth was slower, requiring 15 days to cover the dish. The colony remained white with sparse aerial hyphae and no zonation, though the inoculation site gradually greened, conidia and pigments were absent. On MEA, rapid growth covered the dish in 8 days, yielding a rough-edged colony with abundant aerial hyphae that thickened at distal/lateral margins. Sporulation initiated after 10 days, forming fan-shaped patterns, no odor or pigment was observed.

##### Notes.

Phylogenetically, *Trichoderma yaanensis* strain SICAU 25-0181 and strain SICAU 25-0182 formed a distinct clade and is related to *T. merleae* (MST FP3586) in the Koningii clade, but the similarities of *rpb*2 and *tef*1-α between these two species were only 97.9% and 99.5%, respectively. *Trichoderma yaanensis* is classified within the Koningii clade and is phylogenetically closely related to *T. merleae*. Currently, there is no available morphological description for *T. merleae*, only molecular data have been documented, thereby rendering morphological comparisons infeasible. Phylogenetic analysis reveals that the new taxon, *Trichoderma yaanensis* (SICAU 25-0181), is closely related to *T. merleae* (MST FP3586), with strong statistical support (Fig. 1). However, our strain differs from *T. merleae* in the ITS region amounting to 11.7% (67/572, 32 gap), 2.1% (17/807, 0 gap) differences in *rpb*2, 0.5% (5/921, 0 gap) differences in *tef*1-α. Pairwise nucleotide comparisons further support the distinction of *T. yaanensis*. The PHI test revealed no significant recombination event between our strain and the closely related taxa (Φw = 1.00) (Fig. 2). Therefore, based on the phylogenetic differences, *T. yaanensis* is introduced as a new species within *Trichoderma*.

## ﻿Discussion

Based on ITS, *tef*1-α, and *rpb*2 DNA sequence datasets as well as morphological evidence, this study describes four new species within the genus *Trichoderma*, classifying them as *Trichoderma bashania*, *T. fargesia*, *T. mianyangensis* and *T. yaanensis*. Phylogenetic analysis revealed their distinct genetic relationships and positions within the family *Trichoderma*. Species of this genus are widely distributed globally, and have been reported in various plants, soil, water, as well as in the air. (Liu et al. 2012; Li et al. 2013; Jambhulkar et al. 2024). The discovery of these new species is significant for understanding the species diversity, classification, and geographical distribution of *Trichoderma*.

Bamboo holds extremely high economic, ecological, and cultural values, and the bamboo species that serve as the staple food for giant pandas are imbued with even more unique significance. China is the only country in the world that has wild giant pandas, and the bamboo species that constitute their primary diet are exclusively distributed within this region. The staple bamboo species that giant pandas rely on for food are facing severe challenges, which will lead to food shortages for giant pandas and further accelerate their extinction. Currently, the staple bamboo species for giant pandas are confronted with numerous threats, including global warming, human-induced harvesting of bamboo shoots, grazing, infrastructure development, as well as pests and diseases (Liu et al. 2014; Xiang et al. 2024). Fungi play a crucial role in the ecosystem of the staple bamboo species for giant pandas. They facilitate the growth of the staple bamboo, enhance its resistance to adverse conditions, and participate in the nutrient cycling beneath the bamboo forest. Through mechanisms such as decomposing organic matter, providing nutrients, and promoting the development of bamboo roots, fungi indirectly influence the growth and quality of the bamboo, thereby affecting the food source for giant pandas. In contrast, pathogenic fungi can directly cause the death of the staple bamboo. Research demonstrates that when *Trichoderma* colonizes bamboo as an endophytic fungus, it substantially enhances the plant’s resistance mechanisms. Following bamboo senescence, this versatile fungus transitions to a saprophytic lifestyle, efficiently decomposing lignocellulosic residues and facilitating nutrient cycling within bamboo ecosystems (Bhunjun et al. 2024). Therefore, *Trichoderma* plays a crucial role in the bamboo forest ecosystem. Currently, research on fungi associated with the staple bamboo species for giant pandas is still remains largely unexplored. Therefore, conducting extensive surveys and sampling will contribute to enriching the fungal resources associated with the staple bamboo species for giant pandas.

Among the four newly discovered species, three originate from Sichuan. This phenomenon is primarily due to the fact that the staple bamboo species for giant pandas are predominantly distributed in this region. The increasing discovery of fungal species offers new clues for exploring the biodiversity of this area and also provides crucial information for the conservation of its ecosystems. All these species are distributed within the Koningii clade, a branch that encompasses multiple economically significant species (Han et al. 2023; Xiang et al. 2024). Some of these species are important pathogens, while others have been developed into biological agents. Therefore, further research on these species is necessary.

## Supplementary Material

XML Treatment for Trichoderma bashania

XML Treatment for Trichoderma fargesia

XML Treatment for Trichoderma mianyangensis

XML Treatment for Trichoderma yaanensis

## ﻿Additional information

### Use of AI

No use of AI was reported.

### Author ORCIDs

Xiulan Xu https://orcid.org/0000-0002-6832-5421

Feng Liu https://orcid.org/0000-0003-4580-7169

Chunlin Yang https://orcid.org/0000-0002-3854-8835

### Data availability

All of the data that support the findings of this study are available in the main text.

## References

[B1] Abdel-Mageed HM Barakat AZ Bassuiny RI Elsayed AM Salah HA Abdel-Aty AM Mohamed SA (2022) Biotechnology approach using watermelon rind for optimization of α-amylase enzyme production from *Trichoderma virens* using response surface methodology under solid-state fermentation. Folia Microbiologica 67(2): 253–264. 10.1007/s12223-021-00929-2 34743285

[B2] Abo-Elyousr KA Abdel-Hafez SI Abdel-Rahim IR (2014) Isolation of *Trichoderma* and evaluation of their antagonistic potential against *Alternaria porri*. Journal of Phytopathology 162(9): 567–574. 10.1111/jph.12228

[B3] Bhunjun CS Chen YJ Phukhamsakda C Boekhout T Groenewald JZ McKenzie EHC Francisco EC Frisvad JC Groenewald M Hurdeal VG Luangsa-ard J Perrone G Visagie CM Bai FY Błaszkowski J Braun U de Souza FA de Queiroz MB Dutta AK Gonkhom D Goto BT Guarnaccia V Hagen F Houbraken J Lachance MA Li JJ Luo KY Magurno F Mongkolsamrit S Robert V Roy N Tibpromma S Wanasinghe DN Wang DQ Wei DP Zhao CL Aiphuk W Ajayi-Oyetunde O Arantes TD Araujo JC Begerow D Bakhshi M Barbosa RN Behrens FH Bensch K Bezerra JDP Bilański P Bradley CA Bubner B Burgess TI Buyck B Čadež N Cai L Calaça FJS Campbell LJ Chaverri P Chen YY Chethana KWT Coetzee B Costa MM Chen Q Custódio FA Dai YC Damm U de Azevedo Santiago ALCM De Miccolis Angelini RM Dijksterhuis J Dissanayake AJ Doilom M Dong W Alvarez-Duarte E Fischer M Gajanayake AJ Gené J Gomdola D Gomes AAM Hausner G He MQ Hou L Iturrieta-González I Jami F Jankowiak R Jayawardena RS Kandemir H Kiss L Kobmoo N Kowalski T Landi L Lin CG Liu JK Liu XB Loizides M Luangharn T Maharachchikumbura SSN Makhathini Mkhwanazi GJ Manawasinghe IS Marin-Felix Y McTaggart AR Moreau PA Morozova OV Mostert L Osiewacz HD Pem D Phookamsak R Pollastro S Pordel A Poyntner C Phillips AJL Phonemany M Promputtha I Rathnayaka AR Rodrigues AM Romanazzi G Rothmann L Salgado-Salazar C Sandoval-Denis M Saupe SJ Scholler M Scott P Shivas RG Silar P Souza-Motta CM Silva-Filho AGS Spies CFJ Stchigel AM Sterflinger K Summerbell RC Svetasheva TY Takamatsu S Theelen B Theodoro RC Thines M Thongklang N Torres R Turchetti B van den Brule T Wang XW Wartchow F Welti S Wijesinghe SN Wu F Xu R Yang ZL Yilmaz N Yurkov A Zhao L Zhao RL Zhou N Hyde KD Crous PW (2024) What are the 100 most cited fungal genera? Studies in Mycology 108: 1–411. 10.3114/sim.2024.108.01 PMC11293126 39100921

[B4] Bissett J (1991) A revision of the genus *Trichoderma*. II. Infrageneric classification. Canad. J. Bot 69(11): 2357–2372. 10.1139/b91-297

[B5] Cai F Druzhinina IS (2021) In honor of John Bissett: Authoritative guidelines on molecular identification of *Trichoderma*. Fungal Diversity 107(1): 1–69. 10.1007/s13225-020-00464-4

[B6] Carbone I Kohn LM (1999) A method for designing primer sets for speciation studies in filamentous ascomycetes. Mycologia 91(3): 553–556. 10.1080/00275514.1999.12061051

[B7] Chaverri P Samuels GJ (2004) *Hypocrea*/*Trichoderma* (Ascomycota, Hypocreales, Hypocreaceae): Species with green ascospores. Studies in Mycology 48: 1–116.

[B8] Chomnunti P Hongsanan S Aguirre-Hudson B Tian Q Peršoh D Dhami MK Alias AS Xu J Liu X Stadler M Hyde KD (2014) The sooty moulds. Fungal Diversity 66: 1–36. 10.1007/s13225-014-0278-5

[B9] Hall TA (1999) BioEdit: A user-friendly biological sequence alignment editor and analysis program for windows 95/98/NT. Nucleic Acids Symposium Series 41: 95–98. 10.1021/bk-1999-0734.ch008

[B10] Han WY Wu ZS Zhong ZH Williams J Jacobsen SE Sun ZD Tang Y (2023) Assessing the biosynthetic inventory of the biocontrol fungus *Trichoderma afroharzianum* T22. Journal of Agricultural and Food Chemistry 71(30): 11502–11519. 10.1021/acs.jafc.3c03240 37471583

[B11] Hansen RL Carr MM Apanavicius CJ Jiang PP Bissell HA Gocinski BL Maury F Himmelreich M Beard S Ouellette JR Kouba AJ (2010) Seasonal shifts in giant panda feeding behavior: relationships to bamboo plant part consumption. Zoo Biology 29(4): 470–83. 10.1002/zoo.20280 19862794

[B12] Harman GE Doni F Khadka RB Uphoff N (2021) Endophytic strains of *Trichoderma* increase plants’ photosynthetic capability. Journal of Applied Microbiology 130(2): 529–546. 10.1111/jam.14368 31271695

[B13] Hasan MM Rahman S Kim GH Abdallah E Oh DH (2012) Antagonistic potentiality of *Trichoderma harzianum* towards seed-borne fungal pathogens of winter wheat cv. Protiva in vitro and in vivo. Journal of Microbiology and Biotechnology 22(5): 585–591. 10.4014/jmb.1107.07063 22561850

[B14] He X Hsu WH Hou R Yao Y Xu Q Jiang DD Wang LQ Wang HR (2020) Comparative genomics reveals bamboo feeding adaptability in the giant panda (*Ailuropoda melanoleuca*). ZooKeys 923(923): 141–156. 10.3897/zookeys.923.39665 32292275 PMC7142162

[B15] Helander M Jia R Huitu O Sieber TN Jia J Niemelä P Saikkonen K (2013) Endophytic fungi and silica content of different bamboo species in giant panda diet Symbiosis 61(1): 13–22. 10.1007/s13199-013-0253-z

[B16] Jaklitsch WM Voglmayr H (2015a) Biodiversity of *Trichoderma* (Hypocreaceae) in southern Europe and Macaronesia. Studies in Mycology 80(1): 1–87. 10.1016/j.simyco.2014.11.001 26955191 PMC4779795

[B17] Jambhulkar PP Singh B Raja M Ismaiel A Lakshman DK Tomar M Sharma P (2024) Genetic diversity and antagonistic properties of *Trichoderma* strains from the crop rhizospheres in southern Rajasthan, India. Scientific Reports 14(1): 8610. 10.1038/s41598-024-58302-5 38616195 PMC11016547

[B18] Jayasiri SC Hyde KD Ariyawansa HA Bhat J Buyck B Cai L Dai YC Abd-Elsalam KA Ertz D Hidayat I Jeewon R Jones EBG Bahkali AH Karunarathna SC Liu JK Luangsa-ard JJ Lumbsch HT Maharachchikumbura SSN McKenzie EHC Moncalvo JM Ghobad-Nejhad M Nilsson H Pang KA Pereira OL Phillips AJL Raspé O Rollins AW Romero AI Etayo J Selçuk F Stephenson SL Suetrong S Taylor JE Tsui CKM Vizzini A Abdel-Wahab MA Wen TC Boonmee S Dai DQ Daranagama DA Dissanayake AJ Ekanayaka AH Fryar SC Hongsanan S Jayawardena RS Li WJ Perera RH Phookamsak R de Silva NI Thambugala KM Tian Q Wijayawardene NN Zhao RL Zhao Q Kang JC Promputtha I (2015) The Faces of fungi database: fungal names linked with morphology, molecular and human attributes. Fungal Diversity 74: 3–18. 10.1007/s13225-015-0351-8

[B19] Jin L Wu DF Li CW Zhang AY Xiong YW Wei RP Zhang GQ Yang SZ Deng WW Li T Li B Pan X Zhang ZZ Huang Y Zhang HM He YG Zou LK (2020) Bamboo nutrients and microbiome affect gut microbiome of giant panda. Symbiosis 80(16): 293–304. 10.1007/s13199-020-00673-0

[B20] Katoh K Standley DM (2013) Mafft multiple sequence alignment software version 7: improvements in performance and usability. Molecular Biology and Evolution 30: 772–780. 10.1093/molbev/mst010 23329690 PMC3603318

[B21] Komon-Zelazowska M Bissett J Zafari D Hatvani L Manczinger L Woo S Lorito M Kredics L Kubicek CP Druzhinina IS (2007) Genetically closely related but phenotypically divergent *Trichoderma* species cause green mold disease in oyster mushroom farms worldwide. Applied and Environmental Microbiology 73(22): 7415–7426. 10.1128/AEM.01059-07 17827333 PMC2168202

[B22] Li QR Tan P Jiang YL Hyde KD Mckenzie EH Bahkali AH Wang Y (2013) A novel *Trichoderma* species isolated from soil in Guizhou, *T. guizhouense*. Mycological Progress 12(2): 167–172. 10.1007/s11557-012-0821-2

[B23] Liu YJ Whelen S Hall BD (1999) Phylogenetic relationships among ascomycetes: Evidence from an RNA polymerse II subunit. Molecular Biology and Evolution 16(12): 1799–1808. 10.1093/oxfordjournals.molbev.a026092 10605121

[B24] Liu M Liu J Wang WM (2012) Isolation and functional analysis of Thmfs1, the first major facilitator superfamily transporter from the biocontrol fungus *Trichoderma harzianum*. Biotechnology Letters 34(10): 1857–1862. 10.1007/s10529-012-0972-x 22661043

[B25] Liu C Wang Y Pan K Li W Zhang L Shen X Liu L Den M (2014) Responses of the antioxidant defense system to drought stress in the leaves of *Fargesia denudata* seedlings, the staple food of the giant panda. Russian Journal of Plant Physiology 61(3): 374–383. 10.1134/S1021443714020083

[B26] Lorito M Woo SL Harman GE Monte E (2010) Translational Research on *Trichoderma*: From’omics to the field. Annual Review of Phytopathology 48(1): 395–417. 10.1146/annurev-phyto-073009-114314 20455700

[B27] O’Donnell K Kistler HC Cigelnik E Ploetz RC (1998) Multiple evolutionary origins of the fungus causing Panama disease of banana: Concordant evidence from nuclear and mitochondrial gene genealogies. Proceedings of the National Academy of Sciences of the USA 95: 2044–2049. 10.1073/pnas.95.5.2044 9482835 PMC19243

[B28] Poveda J Hermosa R Monte E Nicolás C (2019) *Trichoderma harzianum* favours the access of arbuscular mycorrhizal fungi to non-host Brassicaceae roots and increases plant productivity. Scientific Reports 9(1): 11650. 10.1038/s41598-019-48269-z 31406170 PMC6690897

[B29] Rambaut A Drummond A (2016) FigTree: Tree figure drawing tool, version 1.4.3. http://tree.bio.ed.ac.uk/software/figtree

[B30] Ran JH Du BB Yue BS (2009) Conservation of the Endangered giant panda *Ailuropoda melanoleuca* in China: successes and challenges. Oryx 43(2): 176–178. 10.1017/S0030605309432010

[B31] Rifai MA (1969) A revision of the genus *Trichoderma*, CMI, Kew, Surrey, United Kingdom, 54 pp.

[B32] Senshu T Miyata K Ohya A Mikogai J Morita M Nakao T Imazu K Fei LS Niu LL Zhao B Yu XM Lu WQ Wang CD Liu XZ Li XB Li MX Lan JC (2014) Procedure and Mechanisms of Bamboo Cell Wall Digestion in the Giant Panda, *Ailuropoda melanoleuca*. Mammal Study 39(4): 219–228. 10.3106/041.039.0405

[B33] Savoie JM Mata G (2003) *Trichoderma harzianum* metabolites pre-adapt mushrooms to *Trichoderma aggressivum* antagonism. Mycologia. 95(2): 191–199. 10.1080/15572536.2004.11833104 21156605

[B34] Shang XT Qin WR Yang B Dai Q Pan H Yang XY Gu XD Yang ZS Zhang ZJ Zhang L (2024) Integrated framework for dynamic conservation of bamboo forest in giant panda habitat under climate change. Journal of Environmental Management 368: 122052. 10.1016/j.jenvman.2024.122052 39128359

[B35] Tarafder E Wenjun Z Karunarathna SC Elgorban AM Huilian M Nan W Zeng X Yong W Tian FH (2024) Unveiling two new species of *Trichoderma* (Hypocreales, Hypocreaceae) that cause green mold disease on *Stropharia rugosoannulata* from Guizhou Province, China. MycoKeys 110: 36–383. 10.3897/mycokeys.110.134154 PMC11607586 39619667

[B36] Taylor JW Jacobson DJ Kroken S Kasuga T Geiser DM Hibbett DS Fisher MC (2000) Phylogenetic species recognition and species concepts in fungi. Fungal Genetics and Biology 31: 21–32. 10.1006/fgbi.2000.1228 11118132

[B37] Tian ZX Liu XH Fan ZY Liu JG Pimm SL Liu LM Garcia C Songer M Shao XM Skidmore A Wang TJ Zhang YK Chang YD Jin XL Gong MH Zhou LG He XB Dang GD Zhu Y Cai Q (2019) The next widespread bamboo flowering poses a massive risk to the giant panda. Biological Conservation 234: 180–187. 10.1016/j.biocon.2019.03.030

[B38] Tomah AA Abd Alamer IS Li B Zhang JZ (2020) A new species of Trichoderma and gliotoxin role: A new observation in enhancing biocontrol potential of *T. virens* against *Phytophthora* capsici on chili pepper. Biological Control 145: 104261. 10.1016/j.biocontrol.2020.104261

[B39] Tripathi P Singh PC Mishra A Chauhan PS Dwivedi S Bais RT Tripathi RD (2013) *Trichoderma*: A potential bioremediator for environmental clean-up. Clean Technologies and Environmental Policy 15(4): 541–550. 10.1007/s10098-012-0553-7

[B40] White TJ Bruns TD Lee SB Taylor JW (1990) Amplification and direct sequencing of fungal ribosomal RNA genes for phylogenetics. PCR protocols: A guide to methods and applications. Academic Press, New York, 315–322. 10.1016/B978-0-12-372180-8.50042-1

[B41] Xiang J Zhang N Li J Zhu Y Cao TY Wang YJ (2024) Unveiling the Hidden Responses: Metagenomic Insights into Dwarf Bamboo (*Fargesia denudata*) Rhizosphere under Drought and Nitrogen Challenges. International Journal of Molecular Sciences 25(19): 10790–10790. 10.3390/ijms251910790 39409119 PMC11477272

[B42] Yang HB Zhang DY Winkler JA Huang QY Zhang YB Wu PH Liu JG Ouyang ZY Xu WH Chen XD Wu DF Zhang JD Songer M (2024) Field experiment reveals complex warming impacts on giant pandas’ bamboo diet. Biological Conservation 294: 110635. 10.1016/j.biocon.2024.110635

[B43] Zhang GZ Yang HT Zhang XJ Zhou FY Wu XQ Xie XY Zhao XY Zhou HZ (2022) Five new species of *Trichoderma* from moist soils in China. MycoKeys 87: 133–157. 10.3897/mycokeys.87.76085 35221753 PMC8873192

[B44] Zhang ZJ Sheppard JK Swaisgood RR Wang G Nie YG Wei W Zhao NX Wei FW (2014) Ecological scale and seasonal heterogeneity in the spatial behaviors of giant pandas. Integrative Zoology 9: 46–60. 10.1111/1749-4877.12030 24447661

[B45] Zhang S Zhang CL Gao ZF Qiu CW Shi SH Chen ZH Ali MA Wang F Wu FB (2023) Integrated physiological and omics analyses reveal the mechanism of beneficial fungal *Trichoderma* sp. alleviating cadmium toxicity in tobacco (*Nicotiana tabacum* L.). Ecotoxicology and Environmental Safety 267: 115631. 10.1016/j.ecoenv.2023.115631 37890251

[B46] Zhao R Mao LJ Zhang CL (2023) Three new species of *Trichoderma* (Hypocreales, Hypocreaceae) from soils in China. MycoKeys 97: 21–40. 10.3897/mycokeys.97.101635 37181496 PMC10170311

[B47] Zhao R Chen KY Mao LJ Zhang CL (2025) Eleven new species of *Trichoderma* (Hypocreaceae, Hypocreales) from China. Mycology 16: 180–209. 10.1080/21501203.2024.2330400 40083403 PMC11899217

[B48] Zheng H Qiao M Lv YF Du X Zhang KQ Yu ZF (2021) New species of *Trichoderma* isolated as endophytes and saprobes from Southwest China. Journal of Fungi 7(6): 467. 10.3390/jof7060467 34207925 PMC8230185

